# Oncometabolites: Unconventional triggers of oncogenic signalling cascades

**DOI:** 10.1016/j.freeradbiomed.2016.04.025

**Published:** 2016-11

**Authors:** Marco Sciacovelli, Christian Frezza

**Affiliations:** Medical Research Council Cancer Unit, University of Cambridge, Hutchison/MRC Research Centre, Box 197, Cambridge Biomedical Campus, Cambridge CB2 0XZ, United Kingdom

**Keywords:** Mitochondria, Cancer, Oncometabolites, FH, SDH, IDH, Fumarate, Succinate, 2-Hydroxyglutarate

## Abstract

Cancer is a complex and heterogeneous disease thought to be caused by multiple genetic lesions. The recent finding that enzymes of the tricarboxylic acid (TCA) cycle are mutated in cancer rekindled the hypothesis that altered metabolism might also have a role in cellular transformation. Attempts to link mitochondrial dysfunction to cancer uncovered the unexpected role of small molecule metabolites, now known as oncometabolites, in tumorigenesis. In this review, we describe how oncometabolites can contribute to tumorigenesis. We propose that lesions of oncogenes and tumour suppressors are only one of the possible routes to tumorigenesis, which include accumulation of oncometabolites triggered by environmental cues.

## Highlights

• Altered metabolism is an emerging aspect of tumorigenesis.
• Damaged mitochondria accumulate metabolites that can activate oncogenic cascades.
• Environmental and nutritional cues trigger the accumulation of these metabolites.

## 1 Background

Cancer is a complex and multifactorial disease. Although its malignant features have been known for centuries, it was not until the advent of modern biology that the molecular determinants of cancer transformation have been elucidated. In 1911, pioneering work from Peyton Rous showed that avian sarcomas were transmissible to other healthy fowls through a filtrate of the tumours devoid of cells [1], [2]. Later on, the agent present in those extracts and responsible for tumour formation was identified as a retrovirus, later called Rous Sarcoma Virus. This important discovery started the field of tumour virology and led to the identification of the oncogene *v-Src* [3], [4], [5], [6], first in retroviruses and then in normal avian DNA [7]. The emerging idea was that cancer was caused by alterations of the genome. Since then, other oncogenes, including *MYC*, *RAS, ERBB, PI3K* [2], [6] and the first tumour suppressor gene *RB1* [8], [9] were discovered. The causative role of *RB1* inactivation in retinoblastoma formation reinforced the concept of cancer initiation driven by genomic alterations. These discoveries led Knudson and colleagues to hypothesise a “multiple-hit” model of tumorigenesis, where multiple genetic alterations are required to achieve full blown transformation [8]. We now know that tumorigenesis requires the acquisition of multiple enabling features, the hallmarks of cancer, among which metabolic rewiring is becoming increasingly recognised [10], [11]. In a seminal paper, Shim et al. showed that Lactate Dehydrogenase A (LDHA) is a target of the proto-oncogene MYC and is required for *MYC-*induced anchorage-independent growth in both human and mouse cellular models [12]. Since then, scientists have uncovered several aspects of the metabolic reprogramming of cancer, and realised that not only dysregulated metabolism is required to sustain proliferation but it also affects tumour microenvironment and the immune response [11].

The discovery that mutations in the metabolic genes Fumarate hydratase *(FH)* [13], Succinate dehydrogenase *(SDH)* [14], [15], [16], [17] *(IDH)* [18], [19], [20], [21] lead to cancer further supported a primary role of metabolic alterations in tumorigenesis. Thanks to these discoveries, a novel paradigm is emerging whereby mitochondrial metabolites that accumulate in these conditions act as oncogenic signalling molecules, becoming bona fide *oncometabolites*. Recent data suggest that reprogramming of cellular metabolism occurs both as direct and indirect consequence of oncogenic mutations and that environmental cues, such as hypoxia, could affect the metabolic phenotype of cancer cells and the abundance of oncometabolites, amplifying oncogenic cascades. In this review we describe the main oncogenic functions of oncometabolites and how their abundance can be affected by genetic mutations and environmental cues.

## 2 TCA cycle enzymes mutations and the emerging paradigm of oncometabolite-driven tumorigenesis

SDH was the first mitochondrial enzyme found mutated in cancer [14]. It was the first time that mutations of a mitochondrial enzyme, once thought to be incompatible with life [22], were linked to tumour predisposition. This and the subsequent discovery of FH mutations in renal cancer catalysed a substantial effort to elucidate the molecular links between mitochondrial dysfunction and tumorigenesis. These major findings are reported below.

### 2.1 Succinate dehydrogenase (SDH)

SDH is an enzyme of the TCA cycle involved in the conversion of succinate to fumarate and a key component of the mitochondrial respiratory chain. This enzyme is composed of four subunits, SDHA, SDHB, SDHC, SDHD; and two assembly factors, SDHF1 and SDHF2 [23]. Mutations in *SDH* are found in familial paraganglioma and pheochromocytoma [14], [15], [16], [17], [24], [25], renal carcinomas [26], T-Cell leukaemia [27], and gastrointestinal stromal tumours [28]. SDH deficiency causes profound metabolic changes. Recent work showed that mouse SDH-deficient cells have a high demand of extracellular pyruvate and utilise glucose-derived carbons for aspartate biosynthesis through pyruvate carboxylation [29], [30]. Interestingly, this metabolic rewiring could be used to selectively target *Sdhb*^-/-^ cells [29].

One of the most striking features of SDH-deficient cells is the accumulation of succinate [31], a metabolite implicated in tumorigenesis and, for this reasons, recently defined an oncometabolite [32]. Among its many functions, succinate is a competitive inhibitor of α-ketoglutarate (aKG)-dependent dioxygenases (aKGDD), a class of enzymes involved in a plethora of biological processes. For instance, succinate inhibits prolyl-hydroxylases (PHDs), aKGDDs involved in the degradation of Hypoxia Inducible Factor (HIF), leading to the aberrant stabilisation of HIFs even when oxygen is abundant, a condition called pseudohypoxia [33]. Succinate inhibits other aKGDDs, including Ten-Eleven Translocation proteins (TETs), enzymes involved in DNA demethylation [34], [35], leading to CpG island hypermethylation [36]. Succinate also causes the inhibition of Histone Lysine Demethylases (KDMs), aKGDDs involved in histone demethylation [34], causing even further epigenetic changes [36], [37]. Interestingly, DNA hypermethylation phenotype was associated with dedifferentiation and increased invasion potential of SDH-deficient tumours [36], [38]. However, the molecular mechanisms behind this phenotypic switch are still under investigation.

### 2.2 Fumarate hydratase

FH is an enzyme of the TCA cycle that converts fumarate to malate. Whilst homozygous FH mutations cause fumaric aciduria, a condition associated with infantile encephalopathy and brain malformations [39], heterozygous *FH* mutations followed by the loss of heterozygosity of the second allele cause Hereditary Leiomyomatosis and Renal Cell Cancer (HLRCC) [13], [40]. *FH* is also mutated in paraganglioma, pheochromocytoma [41], [42], downregulated in sporadic clear cell carcinomas [43] and deleted in neuroblastoma [44]. Cristal structure of human FH showed that clinically-relevant mutations affect evolutionary conserved regions involved in either the catalytic activity or the folding and stability of the protein [45], leading to abnormal accumulation of fumarate [46], [47], [48]. Loss of FH also leads to a complex rewiring of cell metabolism. For instance, FH loss leads to an increased uptake of glutamine that is diverted into haem synthesis and bilirubin excretion to maintain mitochondrial NADH production and mitochondrial potential. Also, the accumulation of fumarate in FH-deficient cells leads to the reversal of the urea cycle enzyme argininosuccinate lyase (ASL), causing the production of argininosuccinate from fumarate and arginine [47], [48]. This metabolic rewiring makes FH-deficient cells auxotrophic for arginine and sensitive to arginine-depriving agents such as arginine deiminase [48]. Interestingly, arginine depletion has been proposed as therapeutic intervention for renal cell carcinoma [49]. However, in this case, arginine auxotrophy is caused by inactivation of argininosuccinate synthase (ASS1), the urea cycle enzyme that converts aspartate and citrulline to argininosuccinate, which is then converted to arginine and fumarate by ASL. Inactivation of ASS1 has been shown to allow the diversion of aspartate from the urea cycle to pyrimidine biosynthesis, favouring tumour growth [50]. It is tempting to speculate that in FH-deficient cells the activity of ASS1 is inhibited by the accumulation of argininosuccinate, leading to increased pyrimidine biosynthesis. Consistent with an increased utilisation of aspartate, the intracellular levels of this metabolite are very low in FH-deficient cells [48]. However, the rate of pyrimidine synthesis is these cells has not been assessed yet.

Fumarate has been implicated in tumorigenesis of HLRCC and, for this reason, included in the list of oncometabolites [51]. Similarly to succinate, fumarate inhibits several aKGDDs, including PHDs, leading to pseudohypoxia [52]. Of note, the non-canonical activation of NF-kB signalling by fumarate also contributes to the pseudohypoxic phenotype in FH-deficient cells [53]. Although pseudohypoxia has been considered an important driver of tumorigenesis, recent data showed that HIFs are dispensable for the formation of benign pre-tumorigenic lesions in Fh1-deficient mice [54]. Therefore, other mechanisms have been proposed to explain fumarate-dependent tumorigenesis. For instance, recent findings identified the Abelson murine leukemia viral oncogene homolog 1 (*ABL-1*) as potential driver in fumarate-dependent tumorigenesis [55]. Interestingly, ABL-1 inhibitors suppress the invasion properties of FH-deficient cells both *in vitro* and *in vivo.* Mechanistically, through ABL-1 activation, fumarate stimulates an antioxidant response mediated by transcription factor NFE2-related factor 2 (NRF2), and a metabolic rewiring through activation of mammalian target of rapamycin (mTOR)-HIF axis [55]. Fumarate was also shown to promote broad epigenetic changes [56], caused by inhibition of histone and DNA demethylases [34], [35]. However, the relevance of these epigenetic changes in tumorigenesis is still under investigation. Finally, fumarate, a mild electrophilic molecule, was demonstrated to react with thiol residues of proteins through a process called *succination* [57], [58], [59], [60]. This post-translational modification is a distinctive feature of FH-deficient tumours and now used for diagnostic purposes [58]. Although several succinated proteins have been identified in FH-deficient cells [60], [61], the biological roles of this process are still under investigation. It was recently reported that succination of mitochondrial Aconitase (ACO2) impairs its enzymatic activity [61] and succination of Kelch-Like ECH-associated protein-1 (KEAP-1) inhibits its negative modulatory effect on the transcription factor NRF2 [54], [62]. Although NRF2 activation has been previously reported as pro-tumorigenic event [63], its role in FH-deficient tumours is still debated. The thiol residue of the antioxidant tripeptide Glutathione (GSH) is also subject of succination [64], [65] and its depletion causes oxidative stress, which induces senescence in primary FH-deficient kidney cells. Of note, genetic ablation of *p21,* a major player in senescence induction, induces transformation of benign cysts in FH-deficient mice indicating the tumour-suppressive role of senescence in FH-mediated tumorigenesis [65]. Besides revealing potential mechanisms of tumorigenesis caused by loss of FH, these works led to the identification of fumarate as important regulator of redox homeostasis, which goes beyond FH deficiency. For instance, Jin et al. showed that fumarate generated within the TCA cycle can regulate the activity of the antioxidant protein Glutathione Peroxidase (GPx), modulating the antioxidant capacity of the cell. Of note, this function of fumarate is not caused by succination but, rather, by the binding of fumarate to the protein [66].

### 2.3 Isocitrate dehydrogenase (IDH1/IDH2)

Isocitrate dehydrogenases (IDHs) are homodimeric enzymes responsible for the reversible oxidative decarboxylation of isocitrate to aKG. Three different isoforms of IDH have been described, which have distinct subcellular compartmentalisation. IDH3 is a NAD^+^-dependent mitochondrial enzyme, core component of the TCA cycle. The other two IDH isoforms (IDH1 and IDH2) are NADP^+^-dependent proteins expressed respectively in cytosol and mitochondria [25], [67]. Heterozygous missense mutations affecting *IDH1* and *IDH2* were found in gliomas [18], [19] and in acute myeloid leukaemia (AML) [20], [21]. At odds with *SDH* and *FH*, *IDH* mutations are gain-of-function mutations and confer to the enzyme the ability to produce the oncometabolite D-2-hydroxyglutarate (D-2HG) [68], [69]. 2HG, the reduced form of aKG, is naturally present in two optic isomers,D-2HG and L-2HG. In normal cells, 2HG is a minor by-product of metabolism and its levels are kept low by the activity of a conserved family of proteins, the L/d-2-hydroxyglutarate dehydrogenases (L2HGDH and D2HGDH) [70], [71], which convert 2HG to aKG. Homozygous germline mutations in these two enzymes are responsible for a severe form of 2HG aciduria characterised by developmental abnormalities and premature death in children [71], [72]. Moreover, mutations in L2HGDH have been associated with brain tumours [73] and, recently, with kidney cancer [74]. Both isomers of 2HG can affect the enzymatic activity of aKGDD. Whilst the role of D-2HG in PHD inhibition and HIF stabilisation is still controversial [75], this metabolite was shown to inhibit both TETs and KDMs [76], [77]. Consistently, *IDH1*-mutant tumours exhibit DNA hypermethylation in gliomas [78] and leukemia [79], and histone hypermethylation [80]. Evidence that *TET2* and *IDH1/2* mutations are mutually exclusive in AML tumours [79] supports the notion that TETs inhibition and the ensuing DNA hypermethylation are instrumental to IDH-driven tumorigenesis. This hypothesis has been recently corroborated by the finding that DNA hypermethylation causes reduced CCCTC-binding factor (CTCF) binding to DNA, leading to aberrant activation of the oncogene Platelet-derived growth factor receptor (*PDGFRA*) [81] in gliomas. Other mechanisms have been proposed to contribute to 2HG-dependent tumorigenesis in IDH-mutant tumours. For instance, 2HG accumulation inhibits ATP Synthase within the mitochondria activating a series of downstream signals that involve mTOR suppression [82]. Regardless the molecular underpinnings, research on IDH-mutant tumours uncovered an unexpected but powerful role of 2HG as oncogenic molecule. In support of this role, it was demonstrated that the incubation of cells with D-2HG promotes cytokine independence and alters differentiation in hematopoietic cells, proving compelling evidence that a small molecule is sufficient to transform cells [83]. Interestingly, the role of 2HG in tumorigenesis goes beyond IDH-mutant tumours. For instance, 2HG accumulation can be driven by a metabolic reprogramming caused by MYC activation in aggressive breast cancer, leading to DNA hypermethylation akin to that observed in IDH mutant tumours [84]. Also, 2HG can be produced by the promiscuous activity of the enzyme Phosphoglycerate Dehydrogenase, another important oncogene in breast cancer [85].

## 3 Succinate, fumarate, and 2HG: overlapping and distinct functions

**Fig. 1:**
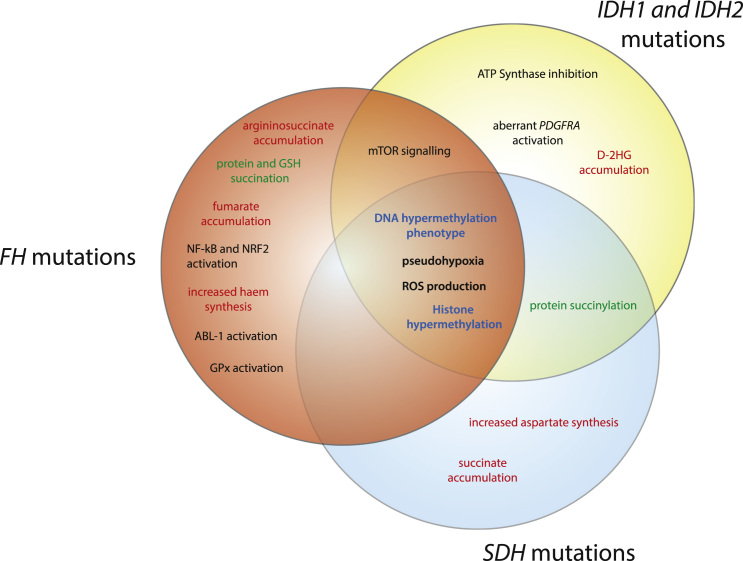
Schematic representation of overlapping features of tumours harbouring *FH*, *SDH*, and *IDH* mutations. Mutations in *FH*, *SDH*, and *IDH* lead to the accumulation of fumarate, succinate, and 2HG, respectively, activating a plethora of signalling cascades. Converging signatures, mainly mediated by aKGDD inhibition, include pseudohypoxia, histone and DNA hypermethylation. The colour of text indicates metabolic alterations (red), epigenetic alterations (blue), post-translational modifications (green) and other pro-tumorigenic alterations (black) elicited by these metabolites. (For interpretation of the references to color in this figure legend, the reader is referred to the web version of this article.)

The brief description above suggests that signalling cascades elicited by fumarate, succinate, and 2HG, share some common targets but also exhibit distinct features, which can explain the different types of tumours associated with their accumulation (Fig. 1). The converging signatures, mostly mediated by aKGDD inhibition, include pseudohypoxia and broad epigenetic changes, such as DNA and histones hypermethylation. Interestingly, recent studies showed that pseudohypoxic genes, besides being regulated by HIFs, are also transcriptionally controlled by TETs. Therefore, epigenetic changes are required for a full pseudohypoxic response triggered by these metabolites. However, these metabolites have different IC_50_ for TETs and KDMs [35], suggesting that their epigenetic effect might have different outcomes, with fumarate being the most effective TETs inhibitor and 2HG the poorest. These metabolites exert other distinct biological functions. As indicated above, fumarate accumulation leads to protein succination, triggering a plethora of biological changes that may synergise with or counteract aKGDDs inhibition. Also, accumulation of 2HG has been shown to increase protein succinylation via inhibition of SDH and subsequent accumulation of succinyl-CoA [86]. This post translation modification leads to a reprogramming of mitochondrial function and induces resistance to apoptosis. Finally, fumarate and 2HG elicit opposite effects on mTOR signalling, with important consequences for the development of specific tumour types [55], [82]. Therefore, although characterised by a very similar chemical structure, succinate, fumarate, and 2HG, appear to have distinct biological roles, well beyond inhibition of aKGDDs.

## 4 Environmental cues regulates oncometabolite production

**Fig. 2:**
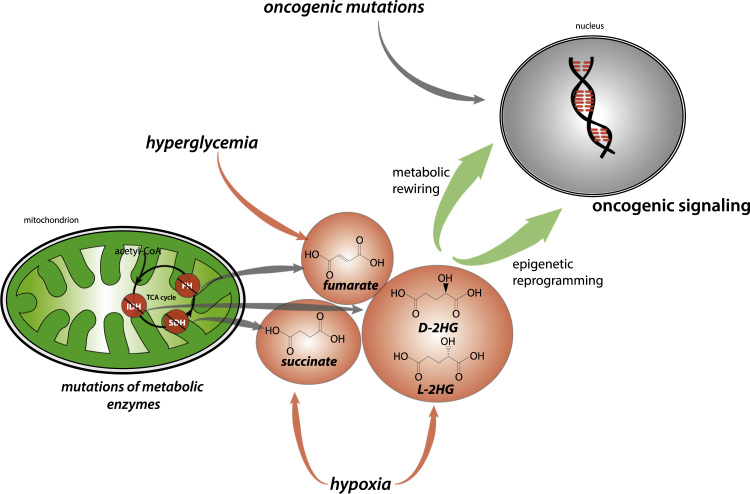
An oncometabolic perspective of tumorigenesis. Schematic representation of how oncometabolite affect the process of tumorigenesis. The indicated oncometabolites can accumulate as a consequence of mutations of TCA cycle enzymes or environmental cues, such as hypoxia or hyperglycemia. These metabolites can act as proper oncogenic triggers and can drive transformation even in the absence of genetic alterations.

The observation that 2HG is sufficient to promote tumorigenesis [83] raised the possibility that environmental cues that increase this metabolite could contribute to tumorigenesis, without underpinning IDH mutations. In support to this hypothesis, it has been shown that hypoxia leads to the production of 2HG (Fig. 2), either via reductive carboxylation [87] or via promiscuous substrate usage of LDHA [88] and Malic Dehydrogenase 1/2 [89]. Since hypoxia is a common feature of solid tumours [90] it is possible that hypoxia-driven production of 2HG elicits (epi)genetic changes that drive or amplify the process of tumorigenesis.

2-HG is not the only oncometabolite whose levels are altered by hypoxia. During ischemia, succinate significantly accumulates in multiple organs and its oxidation is responsible for ROS generation during reperfusion [91]. Succinate was also shown to increase under hypoxic conditions, in cancer cells cultured in 3D scaffolds [92], consistent with its role as hypoxia sensing molecule. Other cues trigger oncometabolite accumulation. For instance, fumarate was shown to accumulate in hyperglycemic conditions [59], [93] (Fig. 2), likely as a consequence of mitochondrial dysfunction caused by glucose accumulation [94], [95]. We hypothesise that fumarate accumulation observed in diabetes [93], [95], could, at least in part, explain the increase cancer risk in these patients. Together, these studies seem to suggest that environmental or nutritional cues may cause dysregulation of mitochondrial function, leading to oncometabolite accumulation in the absence of underpinning oncogenic mutations.

## 5 Future perspectives

Oncometabolites are emerging as key components of the communication between mitochondria and the nucleus. Chronic accumulation of these small molecules triggered by genetic or environmental cues may alter the epigenetic landscape of the cell eliciting oncogenic signalling cascades. This new paradigm of tumorigenesis challenges the role of gene mutations as the exclusive driving mechanism of tumorigenesis and suggests that the latter may be only one of the possible mechanisms that lead to transformation.

## Competing interests

The authors declare no competing interests.

## Authors’ contribution

MS and CF jointly wrote the manuscript.

## Authors’ information

MS is a Research Associate in the laboratory of CF. CF is a group leader at the MRC Cancer Unit, University of Cambridge, Cambridge, UK. MS and CF are funded by an MRC Core Funding to the MRC Cancer Unit.
